# An efficient photocatalyst based on H_5_PMo_10_V_2_O_40_/UiO-66-NH_2_ for direct hydroxylation of benzene to phenol by H_2_O_2_†

**DOI:** 10.1039/d2ra06197j

**Published:** 2022-10-14

**Authors:** Xu Jia, Fuying Wang, Hao Wen, Liuxue Zhang, Shuyan Jiao, Xiulian Wang, Xinyi Pei, Shuzhou Xing

**Affiliations:** School of Materials and Chemical Engineering, Zhongyuan University of Technology Zhengzhou 450007 PR China zhanglx@zut.edu.cn jiaosy@zut.edu.cn +86-731-62506095 +86-731-62506699; School of Energy and Environment, Zhongyuan University of Technology Zhengzhou 450007 PR China

## Abstract

To realize the direct hydroxylation of benzene to phenol by hydrogen peroxide, an efficient photoactive catalyst system was prepared by the recombination of H_5_PMo_10_V_2_O_40_ and UiO-66-NH_2_. The heterpolyacid was uniformly distributed on the UiO-66-NH_2_, and the combination was stable. The composite could effectively photocatalyze the direct hydroxylation of benzene to phenol by H_2_O_2_ in the mixture solution of acetonitrile and acetic acid. The yield and selectivity were 14.08% and 98.8% under the optimum condition, respectively. The performance of the catalyst still maintained well after 5 catalytic cycles. Hence, the investigated catalyst system might be applied in the field of hydroxylation of benzene to phenol.

## Abstract 2

To realize the direct hydroxylation of benzene to phenol by hydrogen peroxide, an efficient photoactive catalyst system was prepared by the recombination of H_5_PMo_10_V_2_O_40_ and UiO-66-NH_2_.

## Graphical abstract

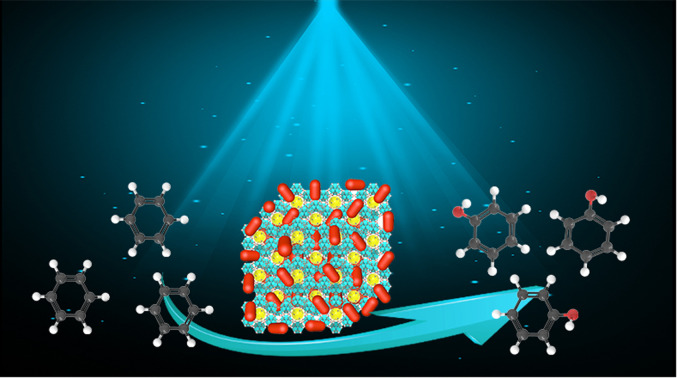

## Introduction

As an important industrial chemical, phenol was applied in the industrial preparation of pharmaceutical products, polymer resins, fungicides, and especially possess widespread application as a preservative.^1,2^ The phenol was traditionally produced from benzene *via* the three-step cumene process, with the disadvantages of low phenol yield, low atom utilization, high energy consumption and formation of equal amount of acetone as the by-product.^3,4^ Currently, the direct hydroxylation of benzene is considered to be an efficient and economical method by using oxidants, such as molecular oxygen (O_2_),^5,6^ nitrous oxide (N_2_O)^7,8^ and hydrogen peroxide (H_2_O_2_).^9,10^ Therein, as its mild use conditions and its environmental friendly by-product water and oxygen, the hydrogen peroxide is considered to be the rising star oxidant in the process of direct catalytic synthesis of phenol from benzene.^11^ A catalyst is needed in the process of direct hydroxylation of benzene with hydrogen peroxide. However, due to the lower efficiency, poorer selectivity and hard recyclability, its extensive use is greatly limited at present.^12^

Based on their porous structures, sufficient stability and chemical resistance, metal–organic frameworks (MOFs) are regarded as facile separation and reusable heterogeneous catalysts in the territory of direct hydroxylation of benzene to phenol.^13^ Fang *et al.* reported a heterogeneous catalyst UiO-66-NH_2_-SA-V prepared by the vanadium oxyacetylacetonate anchored on the Schiff base UiO-66-NH_2_-SA, which demonstrated a high yield and selectivity under the optimized conditions.^14^ Zhang and co-workers reported a Cu^II^-based metal–organic framework, which exhibited a high benzene conversion as well as a high phenol selectivity at 60 °C in water.

Heteropolyacids is a kind of oxygen-containing polyacids, which are composed of heteroatoms (such as P, Si, Fe, Co, *etc.*) and polyatoms (such as Mo, W, V, Nb, Ta, *etc.*) through oxygen atom coordination bridging according to certain structure.^15^ Due to the strong acidity and redox properties, the heteropolyacids is widely concerned as a homogeneous or heterogeneous catalyst.^16^ At present, due to the existence of transition metal, the Keggin-type heteropolyacids with the chemical formula [X^*n*+^M_12_O_40_]^(8−*n*)−^ have been applied as catalysts in the oxidation of organic substrates, especially the hydroxylation of benzene.^17,18^ For instance, Wang group successfully obtained a hybrid catalysts based on pyridine (Py) modified molybdo vanado phosphates, which possessed high catalytic efficiency and high selectivity in the direct hydroxylation of benzene to phenol.^19^

Hence, a novel composite material H_5_PMo_10_V_2_O_40_/UiO-66-NH_2_ was prepared by the electrostatic interaction as a photo-catalyst for direct hydroxylation of benzene to phenol by H_2_O_2_ in this work. Herein, due to the porous structures and sufficient stability, UiO-66-NH_2_ was deemed to be an ideal supporter in the oxidation of organic substrates. The heteropolyacid with Keggin-type showed great potential in catalysis process. The combination of H_5_PMo_10_V_2_O_40_ and UiO-66-NH_2_ would exhibit synergistic effect with relatively high yield and better selectivity in the direct hydroxylation of benzene to phenol, which could be widely used in the field of phenol preparation.

## Experimental

### Materials and synthesis

Zirconium tetrachloride (AR) and 1,4-benzoquinone (97%) was obtained from Guangdong Wong Jiang Chemical Reagent Co., Ltd. 2-Aminoterephthalic acid (AR) and vanadium(v) oxide (AR) was gotten from Zhengzhou Alfa Chemical Co., Ltd. Molybdenum trioxide (99.5%) was purchased from Shanghai Macklin Biochemical Co., Ltd. Phosphoric acid (AR), 30% hydrogen peroxide and *N*,*N*-dimethylformamide (AR) were obtained from Tianjin Kemiou Chemical Reagent Co., Ltd. The other chemicals were commercially obtained and used without further purification.

#### Synthesis of UiO-66-NH_2_

UiO-66-NH_2_ was synthesized from zirconium tetrachloride and 2-aminoterephthalic acid according to our previous work.^20,21^ In brief, zirconium tetrachloride (0.8 mmol) was dispersed into 20 mL of DMF and stirred for 30 min to get a transparent solution. Then, 2-aminoterephthalic acid (0.8 mmol) was added into the mixture to form a homogeneous solution. The mixture was transferred into a 30 mL Teflon-lined stainless steel autoclaves, and 1 equivalent of hydrofluoric acid was added into the solution, heated at 150 °C for 24 h. After cooling to room temperature, the resulting yellow mixtures were collected by centrifugation and washed with DMF and methanol for three times to remove the residual reactants and exchange the DMF. Finally, the obtained solid products were dried in a vacuum oven at 60 °C overnight.

#### Synthesis of H_5_PMo_10_V_2_O_40_

MoO_3_ (7.2 g, 0.05 mol) and V_2_O_5_ (0.91 g, 0.005 mol) was added into 100 mL highly purified water and heated to boiling with stirring. Then, 0.58 g of 85% phosphoric acid (0.005 mmol) was diluted with 10 mL water, titrated into the above-mentioned solution within 30 min, and heated to reflux for 24 hours. The product was collected *via* centrifugation and dried in a vacuum oven at 50 °C. The crude product was recrystallized from water to get orange solid powder.^22,23^

#### Synthesis of H_5_PMo_10_V_2_O_40_/UiO-66-NH_2_ composite

**Scheme 1:**
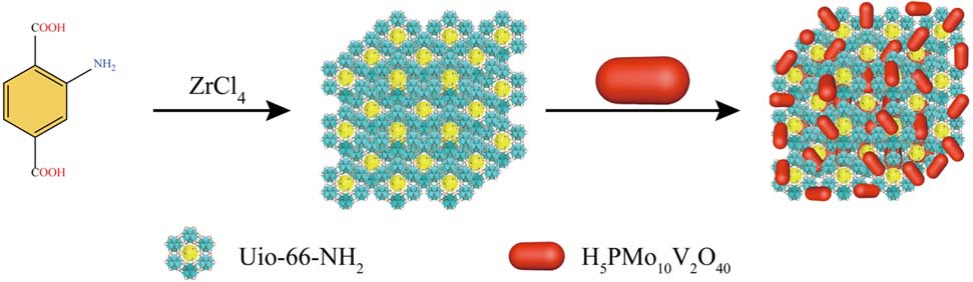
Outline for the synthesis of H_5_PMo_10_V_2_O_40_/UiO-66-NH_2_.

Equivalent amount of H_5_PMo_10_V_2_O_40_ and UiO-66-NH_2_ were transferred into 60 mL CH_2_Cl_2_. The solution was treated with ultrasonic oscillator, and then stirred at room temperature for 24 hours. The composite was washed with ethanol to substitute the residue CH_2_Cl_2_. The orange product was collected *via* centrifugation and dried in a vacuum oven at 50 °C.^24,25^ (Scheme 1)

### Characteristics conditions of photocatalytic hydroxylation

#### Effect of H_2_O_2_ amount to the photocatalytic hydroxylation of benzene

The evaluation of photocatalytic performance was undergone in the quartz reactors. The LED lamp (5 W) was used as the light source. Specifically, H_5_PMo_10_V_2_O_40_/UiO-66-NH_2_ composite (30 mg), acetonitrile (15 mL), acetic acid (3 mL) and benzene (1 mL) was added into the reactor, and gently stirred for a period of time. When the temperature of the solution was increased to 60 °C, the lamp was turned on. Different amounts of the H_2_O_2_ (5.65, 8.48, 11.30, 16.95, 22.60, 33.90 mmol) was titrated into the solution within 30 min. The reaction was maintained for 4 h. Then, the solution was treated by centrifugation. The qualitative and quantitative analysis of the supernatant was performed using toluene as internal standard by gas chromatography-mass spectrometry (GC-MS qp2010) and gas chromatography (Agilent 6820). In parallel research experiments, the best reaction conditions were explored by adjusting the ratio of H_2_O_2_ and benzene, reaction time and solvent.

The conversion of benzene, the selectivity of phenol and the yield of phenol are calculated as follows:
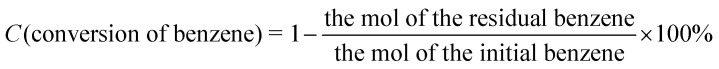

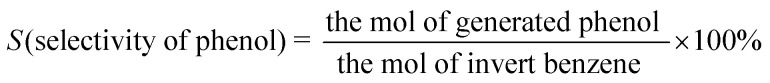

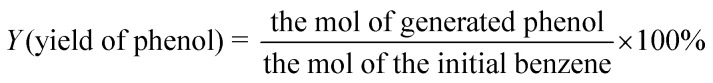


#### Effect of solvent to the photocatalytic hydroxylation of benzene

The experimental procedure was the same as that of effect of H_2_O_2_ amount to the photocatalytic hydroxylation of benzene, except that the solvents used were acetonitrile (15 mL)/acetic acid (3 mL), acetonitrile (18 mL) and acetic acid (18 mL) respectively, and the amount of the H_2_O_2_ used was 6.88 mL in all reactions.

#### Effect of time to the photocatalytic hydroxylation of benzene

The experimental procedure is the same as that of effect of H_2_O_2_ amount to the photocatalytic hydroxylation of benzene, except that the reaction time are 2 h, 4 h and 8 h respectively, the amount of the H_2_O_2_ used was 6.88 mL with the solvent of acetonitrile (15 mL)/acetic acid (3 mL) in all reactions.

#### Photocatalytic reaction mechanism of the photocatalytic hydroxylation of benzene

The experimental procedure is the same as that of effect of H_2_O_2_ amount to the photocatalytic hydroxylation of benzene, except that IPA (1 mM), K_2_Cr_2_O_7_ (1 mM) and EDTA (50 mM) were added into the reactor, respectively. The amount of the H_2_O_2_ used was 6.88 mL in all reactions.

#### The cycle performance of the H_5_PMo_10_V_2_O_40_/UiO-66-NH_2_ composite

The experiment of the catalyst circulation performance was carried out under the optimum reaction conditions. The catalyst was collected by centrifugation at the end of the reaction, washed with ethanol for several times and dried in a vacuum drying oven at 60 °C overnight. Then the recovered catalyst was performed in the next cycle of reaction.

### Characterization of the H_5_PMo_10_V_2_O_40_/UiO-66-NH_2_ composite

The morphology of the composite was tested by scanning electron microscopy (Phenom Prox) with an operating voltage of 10 kV. The X-ray diffraction (Rigaku Uitima IV) pattern was obtained with Cu-Kα radiation at a scan speed of 10 min^−1^ from 5 and 60. N_2_ adsorption–desorption measurements were taken at −196 °C on a JW-BK100C adsorption analytical instrument to determine the specific surface area.

The steady-state photoluminescence was tested on a Hitachi F-7100 spectrofluorometer at room temperature. The transient photocurrent measurements, electrochemistry impedance spectroscopy (EIS) and Mott–Schottky plots were evaluated by the electrochemical workstation (CHI600E). The electrochemical information was collected with the method of standard three-electrode cell: Ag/AgCl electrode (SCE) as the reference electrode, Pt electrode as the counter electrode. The working electrode was prepared with a rectangular FTO glass sheet (2 cm × 1 cm) which was spread 50 μL *N*,*N*-dimethylformamide solution of photocatalysts (10 mg mL^−1^) evenly. The prepared electrode should be dried in the oven at 80 °C for 2 h.

The qualitative analysis of phenol was measured by the gas chromatography-mass spectrometry (GC-MS QP2010). The quantitative analysis of phenol was tested by gas chromatography (Agilent 6820), the injection port temperature was 210 °C, FID was selected as the detector and the temperature was set at 240 °C, the chromatographic column model was Rxi-1ms, the diameter was 0.25 μm, the temperature program of the column: the temperature was kept at 100 °C for 2 minutes, and then, the temperature was raised to 160 °C by the step of 30 °C min^−1^ and maintained for 4 min, then, the temperature was raised to 240 °C at a heating rate of 40 °Cmin^−1^ for 5 min, the retention time of benzene, toluene and phenol were 2.2 min, 2.5 min and 9.8 min, respectively.

## Results and discussion

### Structure characterization of catalysts

**Fig. 1:**
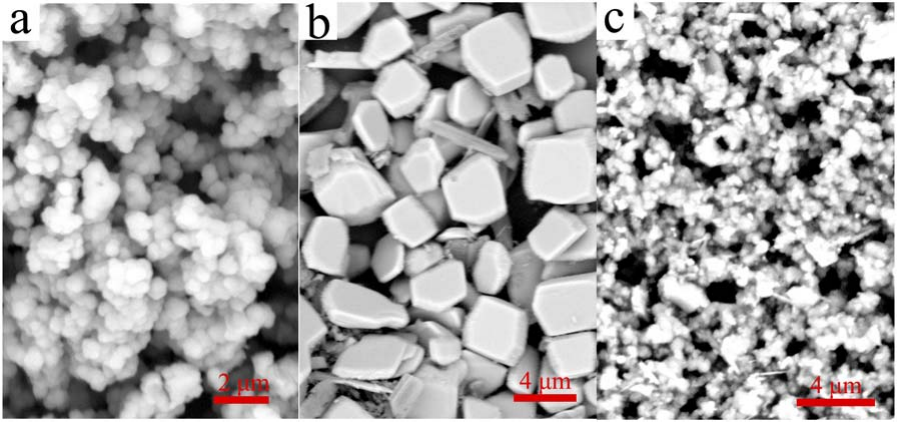
SEM images of the UiO-66-NH_2_ (a), H_5_PMo_10_V_2_O_40_ (b) and H_5_PMo_10_V_2_O_40_/UiO-66-NH_2_ (c).

The main text of the article should appear here with headings as appropriate. The surface morphology of the samples can be observed by the SEM graphs (Fig. 1). The morphology of UiO-66-NH_2_ is mainly composed of spherical and octahedral morphology, which may be caused by the different nucleation rate during the synthesis process, giving rise to the final formation of different crystal morphology.^26^ Except for some crystals with great volume (about 4 μm), the size of most crystals are between tens to hundreds nanometers. As shown in Fig. 1b, the morphology of heteropoly acid is closed to cuboid, and the volume is large and regular, the size is nearly 2 μm. When UiO-66-NH_2_ is compounded with H_5_PMo_10_V_2_O_40_, the size of the composite decreased in some degree. However, the spherical and cuboid structure still existed, which indicated that the composite was successfully synthesized, and the surface structure of the raw materials have not changed after the combination.

**Fig. 2:**
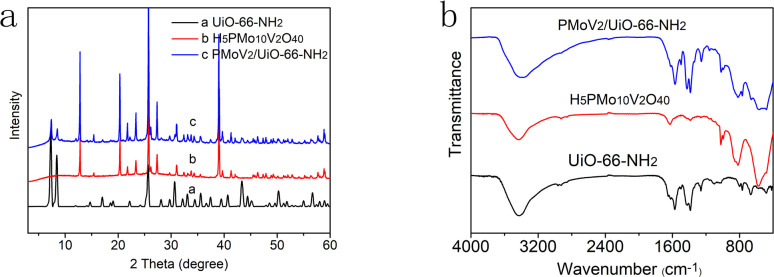
(a) The XRD patterns of UiO-66-NH_2_, H_5_PMo_10_V_2_O_40_ and H_5_PMo_10_V_2_O_40_/UiO-66-NH_2_, and (b) the infrared spectra of UiO-66-NH_2_, H_5_PMo_10_V_2_O_40_ and H_5_PMo_10_V_2_O_40_/UiO-66-NH_2_.

The crystal structure and crystallinity of the catalyst is a key factor of catalytic performance in the reaction process. The XRD spectra of UiO-66-NH_2_, H_5_PMo_10_V_2_O_40_ and H_5_PMo_10_V_2_O_40_/UiO-66-NH_2_ were demonstrated in Fig. 2a. The diffraction peaks of all samples in the whole spectrum are strong and sharp, which proves that the crystallinity is high. The characteristic peaks of the UiO-66-NH_2_ were found at 7.36°, 8.48°, 12.04°, 14.15°, 17.08°, 22.25°, 25.68° and 33.12°, it is consistent with the results reported in the literature.^27^ The characteristic absorption peaks of UiO-66-NH_2_ and H_5_PMo_10_V_2_O_40_ were appeared simultaneously in the XRD spectrum of H_5_PMo_10_V_2_O_40_/UiO-66-NH_2_, which indicated that the composite of H_5_PMo_10_V_2_O_40_/UiO-66-NH_2_ was obtained successfully, and the long-range order of the UiO-66-NH_2_ crystal structure was not changed.^28^ The regular structure is conducive to the transport of reactants and products during the catalytic reaction, which is beneficial to improve the catalytic performance.

The composite process was also revealed by FT-IR analysis (Fig. 2b). The stretching vibrations of the –NH_2_ and –C–C– on aromatic ring skeleton appeared at 3300 cm^−1^ and 1400–1600 cm^−1^ in the infrared spectrum of UiO-66-NH_2_. The characteristic peak of Keggin skeleton appeared at 700–1100 cm^−1^ in the infrared spectrum of H_5_PMo_10_V_2_O_40_. The locations of featured peaks (P–O, 1021 cm^−1^; Mo=O, 996 cm^−1^; Mo–O_b_–Mo, 856 cm^−1^; Mo–O_c_–Mo, 818 cm^−1^) were in well agreement with those in the previous report.^29,30^ The absorption peak of V–O overlaps with that of Mo–O. The scissoring vibration of N–H appeared at 769 cm^−1^ in the infrared spectra of UiO-66-NH_2_ and H_5_PMo_10_V_2_O_40_/UiO-66-NH_2_. From the results of infrared and XRD spectra, it indicated that the H_5_PMo_10_V_2_O_40_/UiO-66-NH_2_ was successfully obtained from UiO-66-NH_2_ and H_5_PMo_10_V_2_O_40_. The peaks appeared at 570 cm^−1^, 678 cm^−1^ and 487 cm^−1^ in the infrared spectrum of H_5_PMo_10_V_2_O_40_/UiO-66-NH_2_ were caused by the skeleton vibration. This phenomenon demonstrated that the skeleton structure of UiO-66-NH_2_ maintained well after it composited with H_5_PMo_10_V_2_O_40_.^31^

The specific surface area was another vital factor of catalytic performance in the reaction process. The parameters were determined from the N_2_ adsorption isotherm of the compounds (Fig. S1 ESI†). The results of BET measurements are listed in Table S1 ESI.† The typical type I isotherm indicated that the H_5_PMo_10_V_2_O_40_/UiO-66-NH_2_ possessed a micropore structure, and the adsorption is mainly through a single-layer adsorption mode.

**Fig. 3:**
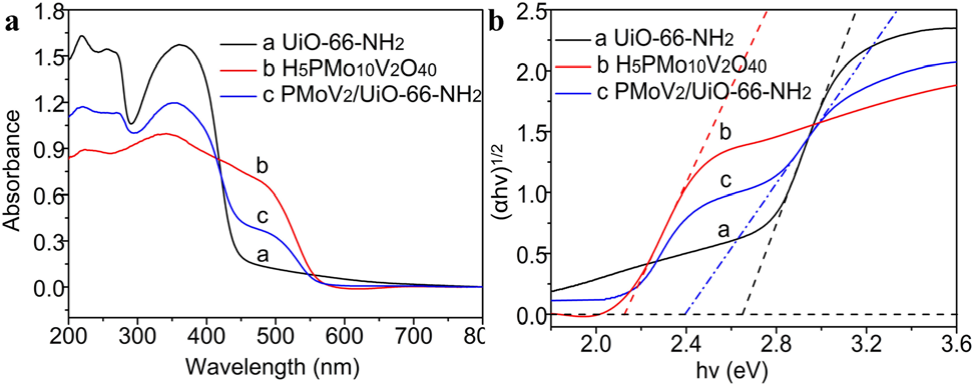
UV-vis diffuse reflectance (a) and band gap (b) of UiO-66-NH_2_, H_5_PMo_10_V_2_O_40_ and H_5_PMo_10_V_2_O_40_/UiO-66-NH_2_.

The photo-response range and energy band of the catalyst depended on the electronic structure (Fig. 3). The photo-response range of UiO-66-NH_2_ is from 200 to 440 nm with two absorption peaks appeared at 220 nm and 360 nm respectively, and the band gap is 2.65 eV. The H_5_PMo_10_V_2_O_40_ has a strong absorption in the visible spectroscopy, the photo-response range is from 200 to 560 nm, and the band gap is 2.12 eV. Compared to Zr-MOFs, the composite H_5_PMo_10_V_2_O_40_/UiO-66-NH_2_ has a broader photo-response range, and the absorption band has a significant red shift, and the band gap was decreased to 2.39 eV. This phenomenon indicated that the heteropolyacids could effectively improve the electronic structure and optical properties of MOFs, and the photocatalytic performance of the composite was enhanced in the direct hydroxylation of benzene to phenol.

**Fig. 4:**
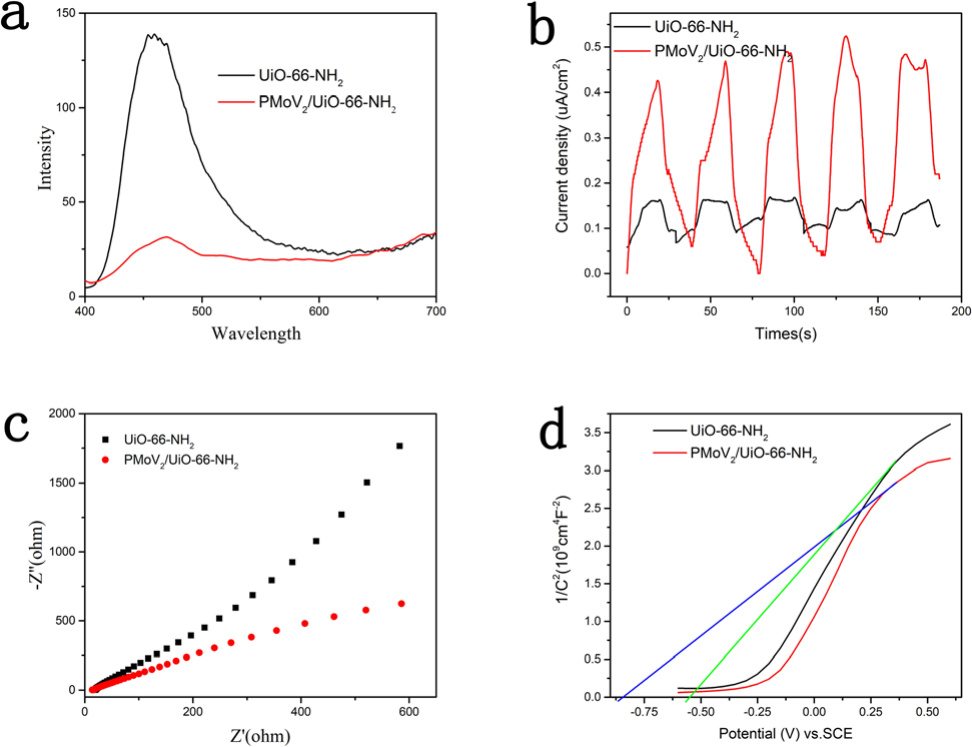
(a) PL spectra for UiO-66-NH_2_ and H_5_PMo_10_V_2_O_40_/UiO-66-NH_2_ with the excitated wavelength of 375 nm. (b) Transient photocurrent response curves of UiO-66-NH_2_ and H_5_PMo_10_V_2_O_40_/UiO-66-NH_2._ (c) EIS spectra for UiO-66-NH_2_ and H_5_PMo_10_V_2_O_40_/UiO-66-NH_2_. (d) Mott–Schottky plots of UiO-66-NH_2_ and H_5_PMo_10_V_2_O_40_/UiO-66-NH_2_.

As a catalyst, the separation efficiency of photo-induced e^−^ and h^+^ was a key parameter for the catalytic performance that could be estimated by the steady-state photoluminescence (PL). The PL intensity of the H_5_PMo_10_V_2_O_40_/UiO-66-NH_2_ was much lower than that of the UiO-66-NH_2_ at the peak of 460 nm, this indicated that the charge separation was enhanced when the introduction of heteropolyacid (Fig. 4a).^32^

Generally, the photocatalytic activity was reflected by the photocurrent intensity, the better photocatalytic activity usually resulted by the higher photocurrent intensity.^33^ Therefore, the separation efficiency of photoinduced charges in the photochemical reactions was characterized by the technique of the transient photocurrent measurement. As shown in Fig. 4b, the composite H_5_PMo_10_V_2_O_40_/UiO-66-NH_2_ possessed a distinctly higher photocurrent density than the UiO-66-NH_2_ at the same conditions. This result revealed the recombination of photogenerated carriers was inhibited after it was compounded with heteropolyacids.

Furthermore, the separation efficiency of photoinduced electron–hole pairs could be reflected by the electrochemical impedance spectroscopy (EIS).^34^ As illustrated in Fig. 4c, the diameter of the H_5_PMo_10_V_2_O_40_/UiO-66-NH_2_ was smaller than that of UiO-66-NH_2_, that indicated the separation of e^−^ and h^+^ was obvious, the catalytic performance of the composites was improved. This result was consistent with the previous detection results of PL and photocurrent intensity.

The information of electronic band structure of photocatalyst could be obtained by Mott–Schottky analysis. As demonstrated in Fig. 4d, the slope of the Mott–Schottky curve of UiO-66-NH_2_ and H_5_PMo_10_V_2_O_40_/UiO-66-NH_2_ were both positive at frequency of 1000 Hz, indicating that the two materials belonged to n-type semiconductors.^35^ Since the flat band potential of the n-type semiconductor is close to its conduction band, it could be estimated from the Mott–Schottky curve that the conduction band positions of UiO-66-NH_2_ and H_5_PMo_10_V_2_O_40_/UiO-66-NH_2_ were −0.55 and −0.84 eV (*vs.* Ag/AgCl), respectively. Compared with UiO-66-NH_2_, the composite H_5_PMo_10_V_2_O_40_/UiO-66-NH_2_ showed a more negative conduction band position, and therefore it could show better photocatalytic performance in the photocatalytic reaction.

### Optimization of photocatalytic performance

**Fig. 5:**
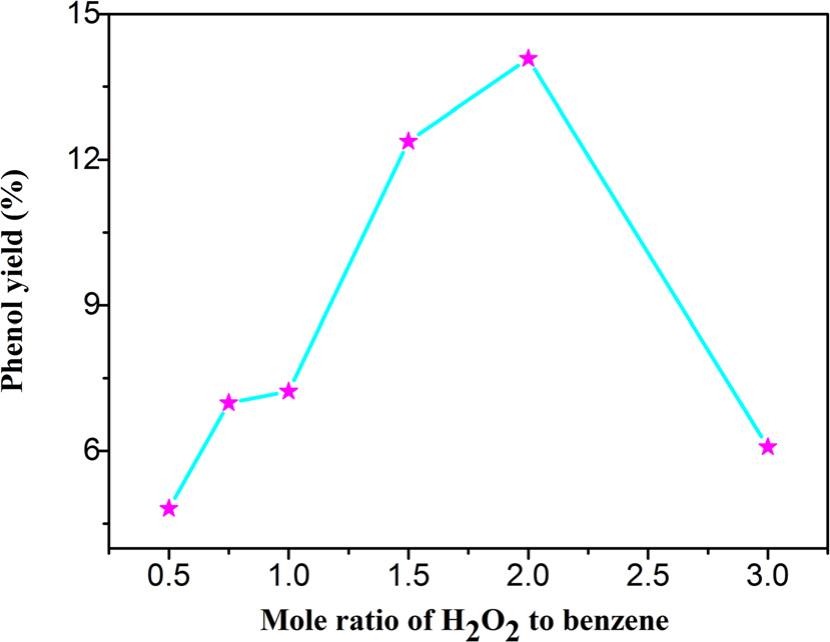
The yield of phenol under different ratios of H_2_O_2_ and benzene.

As the oxidant, the hydrogen peroxide plays a vital role in the photocatalytic hydroxylation of benzene. If without the hydrogen peroxide, it is almost impossible to achieve the goal of the hydroxylation of benzene. In the Table 1 and Fig. 5, it was distinctly demonstrated that the yield of phenol was increased with the increase of H_2_O_2_. When the mole ratio of the H_2_O_2_ and benzene was 2 : 1, the yield of phenol reached the peak of 14.08% with the selectivity of 98.8%. However, if the ratio was greater than 2 : 1, the productivity was decreased. This phenomenon may be caused by the accelerated self-decomposition under a higher concentration. In the controlled experiments, the yields were all relatively low. These results indicated that the catalytic efficiency of the composite was optimal with an appropriate amount of hydrogen peroxide under the visible light.

**Table 1:** Effect of H_2_O_2_ amount to the photocatalytic hydroxylation of benzene

Entry	*n*(H_2_O_2_)/*n*(benzene)	Yield (%)	Selectivity (%)
1	1/2	4.81	99.8
2	3/4	6.99	99.6
3	1/1	7.23	99.4
4	3/2	12.38	99.4
5	2/1	14.08	98.8
6	3/1	6.21	96.4
7^a^	2/1	1.86	99.8
8^b^	2/1	2.09	99.8
9^c^	2/1	—	—

a The UiO-66-NH_2_ used as catalyst instead of H_5_PMo_10_V_2_O_40_/UiO-66-NH_2_.

b Without the light.

c Without the catalyst.

The yield of photocatalytic reaction is also affected by the media. If the catalyst is dispersed well in the solvent, it can promote the combination between reactants and active sites. Zhang and co-workers have discovered the self-decomposition rate of H_2_O_2_ was different, and the self-decomposition of H_2_O_2_ was reduced in the presence of acetic acid. Ahmed *et al.* reported the acetonitrile could react with the H_2_O_2_ to form an intermediate in the photocatalytic hydroxylation of benzene, and the intermediate might be an active oxidant, which was helpful for the formation of phenol. As shown in Table 2, the conversion rate of benzene and the selectivity was 14.08% and 98.8% in the solution of CH_3_CN/CH_3_COOH, respectively. In general, the CH_3_CN/CH_3_COOH mixture was an ideal solvent for the hydroxylation of benzene.

**Table 2:** Effect of solvents to the photocatalytic hydroxylation of benzene

Entry	*n*(H_2_O_2_)/*n*(benzene)	Solvent	Yield (%)	Selectivity (%)
1	2/1	CH_3_CN/CH_3_COOH	14.08	98.8
2	2/1	CH_3_COOH	11.44	99.3
3	2/1	CH_3_CN	13.23	98.9

**Fig. 6:**
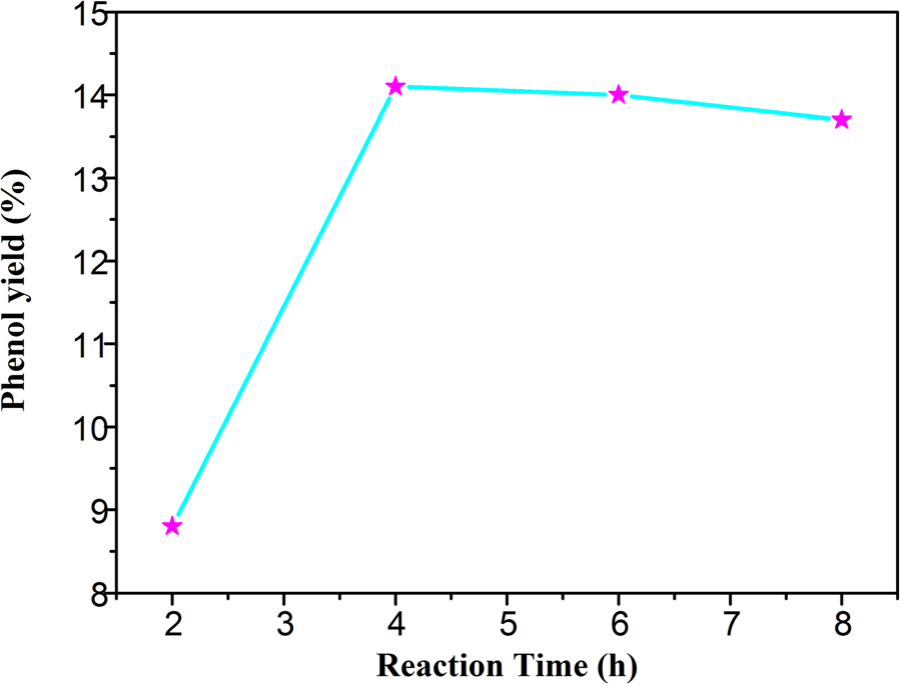
The yield of phenol under different reaction time.

The conversion rate also depends on the reaction time. As shown in Fig. 6, the yield of phenol was increased with the reaction time. The yield was only 8.8% after two hours. The hydroxyl radical increased gradually along with the time, and the amount of phenol also increased. The yield reached 14.08% in 4 hours, and the reaction reached equilibrium. As the activity of phenol is higher than that of benzene, the benzoquinone, a by-product, might be formed by the peroxidation reaction of phenol. In general, the ideal reaction time should be about 4 hours in the photocatalytic hydroxylation of benzene.

**Fig. 7:**
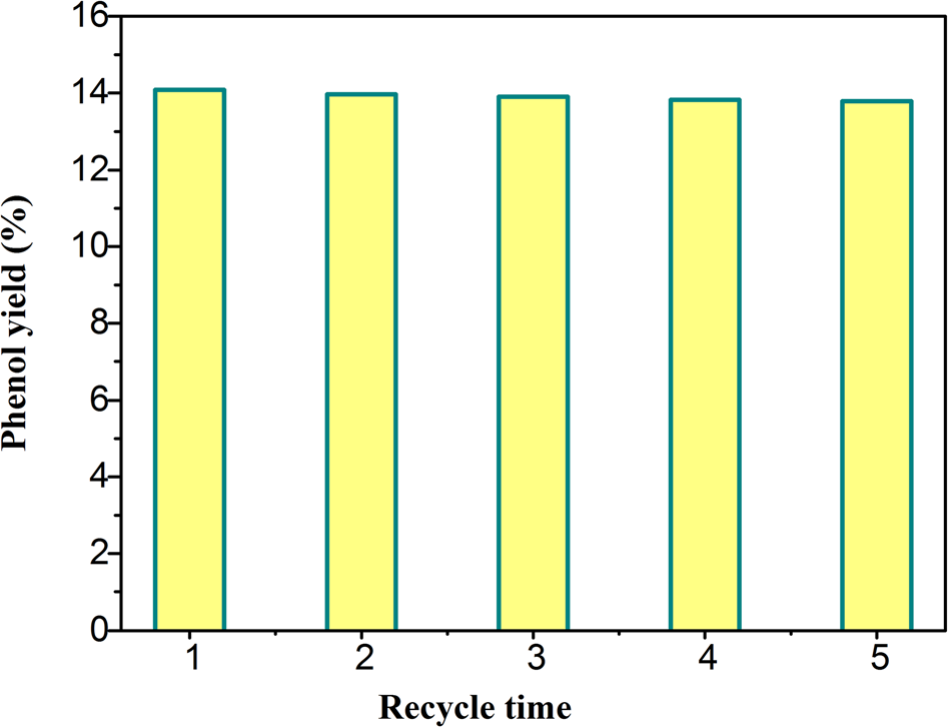
The yield of phenol for the catalyst at different numbers of catalysis cycles.

The reusability of catalyst is an important factor to evaluate the performance of catalyst. The catalytic performance of H_5_PMo_10_V_2_O_40_/UiO-66-NH_2_ was investigated by several cyclic experiments. The conversion rate of benzene was decreased from 14.08% to 13.78% (Fig. 7). This result showed that the performance of the catalyst maintained well after 5 times of recycle use. This phenomenon indicated that the composite was an efficient and stable photocatalyst, and the combination of H_5_PMo_10_V_2_O_40_ and UiO-66-NH_2_ was relatively firm. The catalyst was expected to be applied in the industrial production of phenol.

### Mechanism

**Fig. 8:**
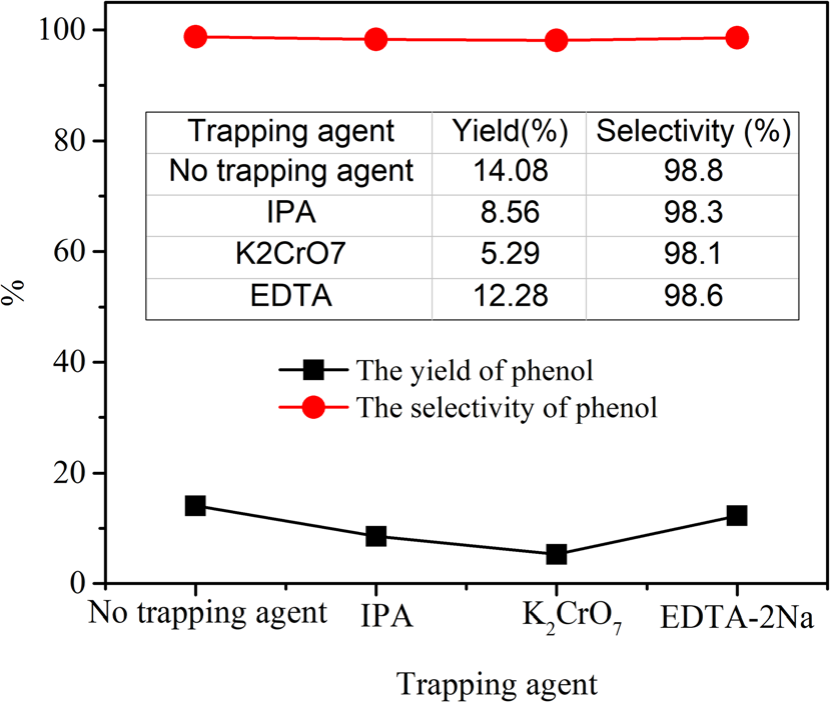
Effects of different scavengers on the photocatalytic hydroxylation of benzene.

It is well known that the ˙OH, h^+^ and e^−^ are vital active species in the photocatalytic reaction.^36^ To explore the key factors in the photocatalytic hydroxylation of benzene, IPA, K_2_Cr_2_O_7_ and EDTA-2Na, were chosen as scavengers to capture the active species including ˙OH, h^+^ and e^−^, respectively. As illustrated in Fig. 8, the yield of phenol was observably reduced in the presence of K_2_Cr_2_O_7_, which could react with e^−^. This result indicated that the electrons played a major role in the formation of phenol. Similarly, when IPA was added to the system, the yield of phenol also decreased significantly, inferring that the ˙OH was another critical factor in this catalytic process. However, the amount of phenol was barely decreased in the presence of EDTA-2Na. In the process of photocatalytic reaction, a large amount of hydrogen peroxide in the system combined with photogenerated electrons to form hydroxyl radical. However, EDTA-2Na could restrain the activity of h^+^ and may also accelerate the recombination of e^−^ and h^+^, hence the formation of ˙O_2_^−^ was also inhibited. These results suggested that the ˙OH and e^−^ as main active substances involved in the photocatalytic reaction of phenol.

Based on the above consequence, a reasonable mechanism was proposed. The H_5_PMo_10_V_2_O_40_/UiO-66-NH_2_ was excited to generate e^−^ and h^+^. The e^−^ was transferred from ˙O_2_^−^ to [VO(O_2_)]^+^ in the V–O clusters to form (VO^2+^). The as-formed (VO^2+^) could reduce H_2_O_2_ to generate active ˙OH *via* a Fenton-like reaction. Then, the ˙OH reacted with the benzene ring to form the hydroxy cyclohexadienyl radical intermediate. Phenol was finally formed when protons were released in the intermediate state.^37^

## Conclusions

In summary, the composite catalyst was prepared by the combination of H_5_PMo_10_V_2_O_40_ and UiO-66-NH_2._ The catalyst showed a high yield and high selectivity of phenol in the direct hydroxylation of benzene by hydrogen peroxide in the mixture solution of acetic acid and acetonitrile. The yield and selectivity were 14.08% and 98.8% in the optimum condition, respectively. The catalytic performance of the composite was maintained well after five cycles. Therefore, due to the efficient catalytic activity and regeneration performance, the composite catalyst showed potential application value in the field of phenol preparation.

## Conflicts of interest

There are no conflicts to declare.

## Supplementary Material

RA-012-D2RA06197J-s001
