# Supplementary material for: An efficient photocatalyst based on H5PMo10V2O40/UiO-66-NH2 for direct hydroxylation of benzene to phenol by H2O2
Source: RSC Adv. 2022 Oct 14;12(45):29433–9. doi: 10.1039/d2ra06197j (PMC9562630; doi:10.1039/d2ra06197j)
Supplement: RA-012-D2RA06197J-s001 [file RA-012-D2RA06197J-s001.pdf]

Electronic Supplementary Material (ESI) for RSC Advances.
This journal is © The Royal Society of Chemistry 2022

Supporting Information

An Efficient Photocatalyst based on $H_5PMo_{10}V_2O_{40}$/UiO-66-NH$_2$ for Direct Hydroxylation of Benzene to Phenol by H_2O_2

Xu Jia [a], Fuying Wang [a], Hao Wen [a], Liuxue Zhang [a*], Shuyan Jiao [a*], Xiulian Wang [b], Xinyi Pei [a], Shuzhou Xing [a]

[1] *School of Materials and Chemical Engineering, Zhongyuan University of Technology, Zhengzhou, 450007, PR China*

[2] *School of Energy and Environment, Zhongyuan University of Technology, Zhengzhou, 450007, PR China*

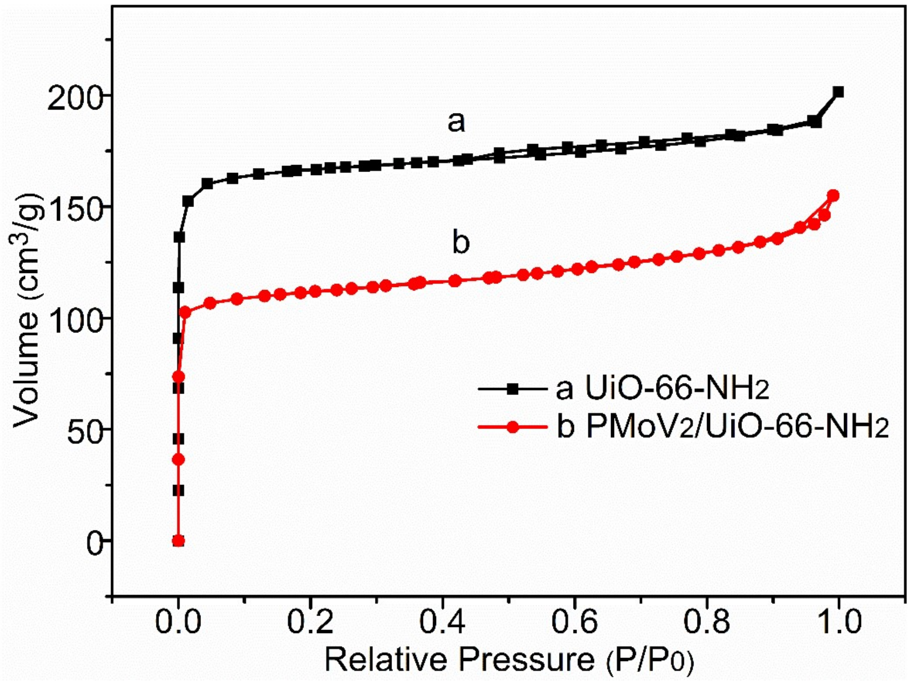

Fig SI1. N_2 adsorption-desorption isotherms of UiO-66-NH$_2$, $H_5PMo_{10}V_2O_{40}$ and $H_5PMo_{10}V_2O_{40}$/UiO-66-NH$_2$.

* Corresponding author. Email address: zhanglx@zut.edu.cn (Liuxue Zhang); jiaosy@zut.edu.cn (Shuyan Jiao).
Tel.: +86 -731-62506699; Fax: +86-731-62506095

Table SI1. The specific surface area, pore volume and pore diameter of $UiO\text{-}66\text{-}NH_2$, $H_5PMo_{10}V_2O_{40}$ and $H_5PMo_{10}V_2O_{40}/UiO\text{-}66\text{-}NH_2$.

sample	S_{BET} (m^2/g)	Pore volume (cm^3/g)	Average pore size (nm)
$UiO\text{-}66\text{-}NH_2$	661	0.405	2.384
$H_5PMo_{10}V_2O_{40}$	9	0.016	2.108
$H_5PMo_{10}V_2O_{40}/UiO\text{-}66\text{-}NH_2$	512	0.285	2.019
